# Structural and Magnetic Studies on Nickel(II) and Cobalt(II) Complexes with Polychlorinated Diphenyl(4-pyridyl)methyl Radical Ligands

**DOI:** 10.3390/molecules26185596

**Published:** 2021-09-15

**Authors:** Ryota Matsuoka, Tatsuhiro Yoshimoto, Yasutaka Kitagawa, Tetsuro Kusamoto

**Affiliations:** 1 Institute for Molecular Science, 5-1 Higashiyama, Myodaiji, Okazaki 444-8787, Aichi, Japan; matsuoka@ims.ac.jp; 2 SOKENDAI (The Graduate University for Advanced Studies), Shonan Village, Hayama 240-0193, Kanagawa, Japan; 3 Department of Materials Chemistry, Ryukoku University, Otsu 520-2194, Shiga, Japan; tatu21307@icloud.com; 4 Division of Chemical Engineering, Department of Materials Engineering Science, Graduate School of Engineering Science, Osaka University, Toyonaka, Osaka 560-8531, Japan; kitagawa@cheng.es.osaka-u.ac.jp; 5 JST-PRESTO, 4-1-8, Honcho, Kawaguchi 332-0012, Saitama, Japan

**Keywords:** ferromagnetic interaction, radical, heterospin

## Abstract

New magnetic metal complexes with organic radical ligands, [M(hfac)_2_(PyBTM)_2_] (M = Ni^II^, Co^II^; hfac = hexafluoroacetylacetonato, PyBTM = (3,5-dichloro-4-pyridyl)bis(2,4,6-trichlorophenyl)methyl radical), were prepared and their crystal structures, magnetic properties, and electronic structures were investigated. Metal ions in [M(hfac)_2_(PyBTM)_2_] constructed distorted octahedral coordination geometry, where the two PyBTM molecules ligated in the *trans* configuration. Magnetic investigation using a SQUID magnetometer revealed that *χT* increased with decreasing temperature from 300 K in the two complexes, indicating an efficient intramolecular ferromagnetic exchange interaction taking place between the spins on PyBTM and M with *J/k*_B_ of 21.8 K and 11.8 K for [Ni^II^(hfac)_2_(PyBTM)_2_] and [Co^II^(hfac)_2_(PyBTM)_2_]. The intramolecular ferromagnetic couplings in the two complexes could be explained by density functional theory calculations, and would be attributed to a nearly orthogonal relationship between the spin orbitals on PyBTM and the metal ions. These results demonstrate that pyridyl-containing triarylmethyl radicals are key building blocks for magnetic molecular materials with controllable/predictable magnetic interactions.

## 1. Introduction

Molecular materials with magnetic functions have been focused on as promising components for future molecule-based devices. This is mainly due to their good tunability in their physical properties by molecular design. Among the molecule-based magnetic materials developed to date, the coupling of magnetic metal ions and open-shell organic radical ligands has resulted in a variety of unprecedented materials such as a one-dimensional chain magnet with slow magnetic relaxation, light-responsive breathing crystals, and high-temperature molecular magnets [1,2,3]. In these materials, precise control of magnetic interactions between the radical and metals is the crucial factor for achieving desired functions. In general, a radical–metal magnetic interaction is interpreted by overlapping and orthogonality of spin orbitals on the radical and metal ion; the former and the latter result in antiferromagnetic (AFM) and ferromagnetic (FM) interactions, respectively [4,5]. In radical-coordinated metal complexes, coordination geometry around the metal center predominantly affects the strength (*J*, an exchange coupling constant) and sign (FM or AFM) of through-bond radical–metal interaction.

Polychlorinated triarylmethyl (PTM) radicals are an important class of stable organic radicals possessing an unpaired electron with *S* = 1/2 [6]. PTM radicals bearing metal-binding sites such as carboxylato and sulfonato moieties have been utilized to prepare magnetic molecular materials using a metal–radical heterospin strategy [7,8]. For example, efficient AFM interactions mediated through the carboxylato groups were elucidated between carboxylato-functionalized PTM, PTMMC, and Cu^II^ with a *J*_R-Cu_/*k*_B_ value of −23.1 K [9].

**Figure 1:**
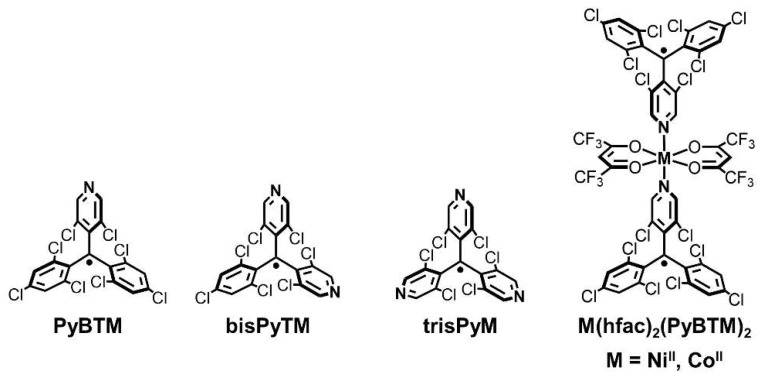
Chemical structures of radicals and [M(hfac)_2_(PyBTM)_2_] (M = Ni^II^, Co^II^).

We have recently prepared a series of PTM radicals, PyBTM, bisPyTM, trisPyM, (PyBTM = (3,5-dichloro-4-pyridyl)bis(2,4,6-trichlorophenyl)methyl radical, bisPyTM = bis(3,5-dichloro-4-pyridyl)(2,4,6-trichlorophenyl)methyl radical, trisPyM = tris(3,5-dichloro-4-pyridyl)methyl radical), possessing one, two, and three pyridyl groups in the triarylmethyl skeleton (Figure 1) [10,11,12]. Importantly, the pyridyl nitrogen atoms in these radicals can coordinate to metal ions such as Cu^II^, Mn^II^, Zn^II^, and Au^I^ ions [12,13,14,15,16,17,18]. We have shown that Cu^II^ complexes [Cu^II^(hfac)_2_(PyBTM)_2_] (hfac = hexafluoroacetylacetonato) and [Cu^II^(hfac)_2_(bisPyTM)] demonstrated FM radical–Cu^II^ interaction with *J*_R-Cu_/*k*_B_ of 47 K for the former and 46 K (low-temperature phase) and 11 K (high-temperature phase) for the latter [15,16], while [Mn^II^(hfac)_2_(PyBTM)_2_] displayed AFM radical–Mn^II^ coupling (*J*_R-Mn_/*k*_B_ of −9.7 K) [13]. These efficient radical–metal couplings are attributed to the spin densities of PyBTM and bisPyTM, which are well delocalized onto the π-conjugated framework including the pyridyl nitrogen atoms [10,11].

In this study, we prepared novel Ni^II^ and Co^II^ complexes with coordinated PyBTMs, [M(hfac)_2_(PyBTM)_2_] (M = Ni^II^, Co^II^) (Figure 1), to elucidate how the central metal ion affects structural and magnetic characteristics in this class of molecular heterospin systems. We herein report the synthesis, structures, and magnetic properties of [M(hfac)_2_(PyBTM)_2_] (M = Ni^II^, Co^II^), where efficient FM radical–metal couplings were achieved. These results are different from PTM-ligated Co^II^ and Ni^II^ complexes [M(PTMMC)_2_(pyridine)_3_(H_2_O)] (M = Ni^II^, Co^II^) reported previously, where two PTMMCs were coordinated to the metal ions via the carboxylate groups in the trans fashion to demonstrate AFM radical–metal couplings with *J*_R-M_/*k*_B_ = −23.6 K for M = Ni^II^ and −7.6 K for M = Co^II^ [19].

## 2. Results and Discussion

**Figure 2:**
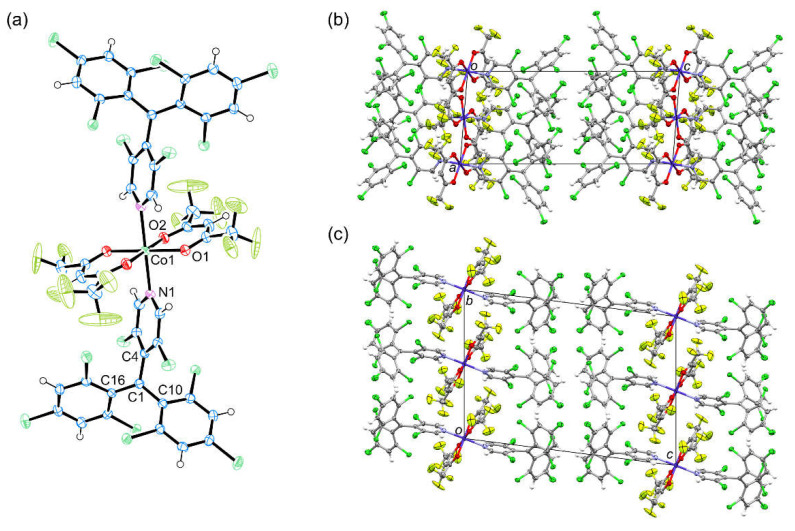
(**a**) Molecular structure of [Co^II^(hfac)_2_(PyBTM)_2_] in the crystal. (**b**) Crystal structure viewed along the *b*-axis and (**c**) the *a*-axis. The disorder of trifluoromethyl groups is omitted for clarity.

[M(hfac)_2_(PyBTM)_2_] (M = Ni^II^, Co^II^) were prepared by mixing PyBTM and M(hfac)_2_·nH_2_O in mixed solvent under reflux. The resulting complexes were characterized by elemental analysis, diffuse reflectance spectroscopy, and powder and single-crystal X-ray diffractions (Figure S1–3, Table S1). The complexes showed no detectable ESR signal in the solid state at room temperature. Both the X-ray diffraction studies revealed that [Ni^II^(hfac)_2_(PyBTM)_2_] and [Co^II^(hfac)_2_(PyBTM)_2_] are both isostructural with the other [M(hfac)_2_(PyBTM)_2_] (M = Zn^II^, Cu^II^, Mn^II^) reported previously [13,16]. Here, we mainly focus on the crystal structure of [Co^II^(hfac)_2_(PyBTM)_2_] as the representative to discuss the structural characteristics of the two complexes. The unit cell contains two crystallographically non-equivalent [Co^II^(hfac)_2_(PyBTM)_2_] molecules, and one of them is shown in Figure 2a. The Co^II^ ion forms a distorted octahedral coordination geometry, and is located on an inversion center. Two PyBTM radicals ligate to the Co^II^ ion via the nitrogen atoms in a *trans* configuration. Averaged M–O1, M–O2, and M–N bond lengths for the two non-equivalent complexes are 2.048 Å, 2.047 Å, and 2.140 Å for M = Co^II^ and 2.028 Å, 2.040 Å, and 2.078 Å for M = Ni^II^. The coordination geometry is elongated along the M–N bond direction, as observed similarly in [Zn^II^(hfac)_2_(PyBTM)_2_] and [Mn^II^(hfac)_2_(PyBTM)_2_] [13,16]. Trifluoromethyl groups in the hfac ligands are disordered in two positions. In the ligated PyBTM radicals, the C1 atom is located within a plane constructed by C4, C10, and C16 atoms, forming sp^2^ hybridization. The three aromatic rings construct propeller-like structures due to steric repulsion between the chlorine atoms in their *ortho* positions. These structural characteristics are similar to a non-coordinated PyBTM [10], confirming the radical character of the PyBTM ligands in [M(hfac)_2_(PyBTM)_2_].

Shortest M–M and C1–C1 distances in the crystal lattice are 8.5382 Å and 8.501(6) Å for M = Co^II^ and 8.5557 Å and 8.51(2) Å for M = Ni^II^; the spin centers are separated sterically. Several intermolecular Cl···F, Cl···H, and F···F atomic contacts were confirmed, which would mediate weak intermolecular AFM interactions suggested in the magnetic studies.

**Figure 3:**
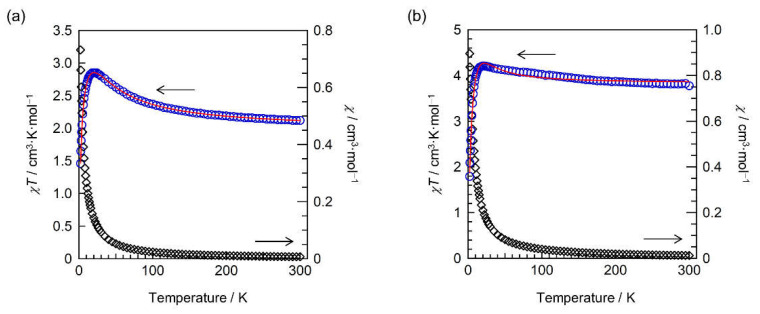
Temperature-dependent *χ* and *χT* at 10 kOe of (**a**) [Ni^II^(hfac)_2_(PyBTM)_2_] and (**b**) [Co^II^(hfac)_2_(PyBTM)_2_]. Red lines indicate fitting curves.

The magnetic properties of the complexes were examined using a SQUID magnetometer (Figure 3a). In [Ni^II^(hfac)_2_(PyBTM)_2_], the *χT* value at 300 K was 2.12 cm^3^·K·mol^−1^ and was a little higher than the value expected from one *S* = 1 for Ni^II^ and two *S* = 1/2 spins for PyBTMs (1.75 cm^3^·K·mol^–1^ with *g* = 2.00). The *χT* value increased with decreasing temperature and reached a maximum around 20 K, indicating an FM interaction dominant between the spins. The subsequent decrease in the *χT* value below 20 K would be due to the intermolecular AFM interaction, as observed similarly in the other [M(hfac)_2_(PyBTM)_2_] complexes [13,16]. The FM interaction can be attributed to the intramolecular exchange interaction between the Ni^II^ and the PyBTM spins. The temperature dependence of *χT* was analyzed by a symmetrical three-spin model with *H* = −2*J*_M-R_(*S*_R_*S*_M_ + *S*_M_*S*_R_) [20], where *S*_M_ = 1 or 3/2 for M = Ni^II^ or Co^II^, *S*_R_ = 1/2, and *J*_M-R_ indicates an exchange coupling constant between the spins on M and PyBTM. The *χT*–*T* plot of [Ni^II^(hfac)_2_(PyBTM)_2_] was fitted using Equation (1) [20]. *N*, *μ*_B_, and *k*_B_ indicate the Avogadro constant, the Bohr magneton, and the Boltzmann constant, respectively; *x* = exp(*J*_M-R_/*k*_B_*T*), and *θ* represents the intermolecular AFM interactions.

*χ* = [*Nμ*_B_^2^*g*^2^/*k*_B_(*T*−*θ*)][(10 + 2*x*^−2^ + 2*x*^−4^)/(5 + 3*x*^−2^ + 3*x*^−4^ + *x*^−6^)] (1)

The fitting afforded *J*_Ni-R_/*k*_B_, *g*, and *θ* values of 21.8 K, 2.13, and −2.63 K, respectively. The positive *J*_Ni-R_/*k*_B_ value confirms an efficient FM PyBTM–Ni^II^ interaction.

Figure 3b depicts the temperature dependence of *χT* of [Co^II^(hfac)_2_(PyBTM)_2_] measured at 10 kOe. The *χT* value was 3.78 cm^3^·K·mol^−1^ at 300 K (2.625 cm^3^·K·mol^−1^ when assumed from isolated one *S* = 3/2 and two *S* = 1/2 spins with *g* = 2.00), and increased upon cooling, confirming the predominant FM interaction taking place between the spins on Co^II^ with *S* = 3/2 and PyBTM with *S* = 1/2. The subsequent decrease in *χT* below 20 K is presumably due to the intermolecular AFM interaction. The *χT* behavior was analyzed with Equation (2) based on the symmetrical three-spin model, as was done for [Ni^II^(hfac)_2_(PyBTM)_2_], to yield *J*_Co-R_/*k*_B_, *g*, and *θ* values of 11.8 K, 2.24, and −3.87 K, respectively. A *g*-value larger than 2.0 would reflect magnetic anisotropy and spin-orbit coupling characteristic of Co^II^ [20]. The positive *J*_Co-R_/*k*_B_ value agreed with the presence of FM interaction between the spins.

*χ* = [*Nμ*_B_^2^*g*^2^/4*k*_B_(*T*−*θ*)][(35 + 10*x*^−3^ + 10*x*^−5^ + *x*^−8^)/(3 + 2*x*^−3^ + 2*x*^−5^ + *x*^−8^)] (2)

The magnetic couplings between metal ions and radicals in the present heterospin system are interpreted based on the overlap or orthogonality of relevant spin orbitals, in which the former and the latter mediate AFM and FM exchange interactions, respectively. As the SOMO of PyBTM on the nitrogen atom has a pπ character, orbital overlap is expected for d_yz_ and d_xz_ orbitals of the metal ions while an orthogonal relationship can be achieved for d_z2_, d_x2__–y2_, and d_xy_ orbitals, assuming that the M–N bond direction corresponds to the z-axis direction. All these orbital interactions contribute to the magnetism of the complexes, thereby determining their net magnetic properties. In [M(hfac)_2_(PyBTM)_2_], it is expected that Ni^II^ possesses two unpaired electrons on the d_z2_ and d_x2__–y2_ orbitals while Co^II^ bears two of the three unpaired electrons on the d_z2_ and d_x2__–y2_ orbitals. Accordingly, the FM couplings observed experimentally can be explained as orbital orthogonality of the spin orbitals.

**Figure 4:**
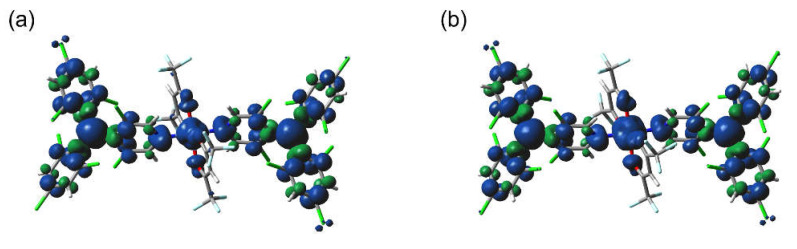
Spin-density distribution of (**a**) [Ni^II^(hfac)_2_(PyBTM)_2_] and (**b**) [Co^II^(hfac)_2_(PyBTM)_2_] calculated using DFT: uB3LYP/6-31G(d) for C, H, O, Cl, F, and N; LANL2DZ for Ni and Co. One of the two crystallographically independent molecules is shown in each complex.

The FM radical–metal couplings in [M(hfac)_2_(PyBTM)_2_] were reproduced theoretically by broken-symmetry DFT calculation. The geometries used for the calculations were extracted from the crystallographic data. [Ni^II^(hfac)_2_(PyBTM)_2_] possesses a quintet ground state. The phase of spin density on the centering carbon atoms of the PyBTM ligands and the Ni^II^ ion is the same (Figure 4a). The results represent an FM configuration of the spins. The calculated *J*_Ni–R_/*k*_B_ values are 27.9 and 29.8 K for the two crystallographically non-quivalent molecules. These results were consistent with the experimental results, while the *J*_Ni–R_/*k*_B_ values calculated are little larger than the value estimated experimentally. Similarly, sextet ground states with FM radical–Co^II^ couplings (5.4 and 4.8 K) are calculated for two crystallographically independent [Co^II^(hfac)_2_(PyBTM)_2_] molecules. The spin density distribution of one of the two [Co^II^(hfac)_2_(PyBTM)_2_] molecules is shown in Figure 4b. Namely, the theoretical calculations nicely support the FM interaction detected in magnetic studies.

In conclusion, novel heterospin complexes [Ni^II^(hfac)_2_(PyBTM)_2_] and [Co^II^(hfac)_2_(PyBTM)_2_] were prepared and their structural and magnetic characteristics were investigated. The metal ions formed a distorted octahedral coordination environment with the two PyBTM radicals coordinating in the *trans* configuration in the solid state. Magnetic investigation elucidated FM interactions operated between PyBTM and the metal ions in the two complexes. The FM interactions could be interpreted as an orthogonal relationship between the spin orbitals. These results demonstrate that pyridyl-containing triarylmethyl radicals are key building blocks for magnetic molecular materials with controllable/predictable magnetic interactions.

## 3. Materials and Methods

### 3.1. General Methods

Unless otherwise noted, solvents (dichloromethane, chloroform, and hexane) and reagents ([Co^II^(hfac)_2_·nH_2_O] and [Ni^II^(hfac)_2_·nH_2_O]) were purchased from TCI Co., Ltd. (Tokyo, Japan), FUJIFILM Wako Pure Chemical Corp. (Osaka, Japan), Kanto Chemical Co., Inc. (Tokyo, Japan), or Merck KGaA (Darmstadt, Germany), and used without further purification. Dry dichloromethane and hexane were purified with Ultimate Solvent System 4S (AS ONE Corp., Osaka, Japan). PyBTM was prepared according to the previous literature [10].

### 3.2. Syntheses of Complexes

*[Co^II^(hfac)_2_(PyBTM)_2_]*. Under a nitrogen atmosphere, [Co^II^(hfac)_2_·nH_2_O] (51.7 mg, 0.102 mmol for *n* = 2) was dissolved in dry hexane (30 mL) under reflux. To this solution was added a solution of PyBTM (105 mg, 0.202 mmol) in dry dichloromethane and dry hexane (1.4 mL and 2.6 mL) at the same temperature, and the mixture was refluxed for 16 h. The resulting suspension was cooled to −30 °C for 2 h, and the precipitates were collected by filtration. The solid was recrystallized from chloroform to give [Co^II^(hfac)_2_(PyBTM)_2_] as a red solid (37.9 mg, 0.025 mmol, 25%). Anal. Calcd for C_46_H_14_Cl_16_F_12_N_2_O_4_Co ([Co^II^(hfac)_2_(PyBTM)_2_]): C, 36.52; H, 0.93; N, 1.85. Found: C, 36.48; H, 1.17; N, 1.91. The single crystal of [Co^II^(hfac)_2_(PyBTM)_2_] suitable for X-ray analysis was obtained by slow vapor diffusion of hexane into a chloroform solution of [Co^II^(hfac)_2_(PyBTM)_2_] at 30 °C.

*[Ni^II^(hfac)_2_(PyBTM)_2_]*. Under a nitrogen atmosphere, [Ni^II^(hfac)_2_·nH_2_O] (51.4 mg, 0.101 mmol for *n* = 2) was dissolved in a mixed solvent of dry hexane (30 mL), dry dichloromethane (15 mL), and dry chloroform (14 mL) under reflux. To this solution was added a solution of PyBTM (105 mg, 0.202 mmol) in dry dichloromethane and dry hexane (1.4 mL and 2.6 mL) at the same temperature, and the mixture was refluxed for 15 h. The resulting suspension was cooled to −30 °C for 2 h, and the precipitates were collected by filtration. The solid was recrystallized from chloroform to give [Ni^II^(hfac)_2_(PyBTM)_2_] as a red solid (71.4 mg, 0.047 mmol, 47%). Anal. Calcd for C_46_H_14_Cl_16_F_12_N_2_O_4_Ni ([Ni^II^(hfac)_2_(PyBTM)_2_]): C, 36.53; H, 0.93; N, 1.85. Found: C, 36.28; H, 1.03; N, 1.90. The single crystal of [Ni^II^(hfac)_2_(PyBTM)_2_] suitable for X-ray analysis was obtained by slow vapor diffusion of hexane into a chloroform solution of [Ni^II^(hfac)_2_(PyBTM)_2_] at 30 °C.

### 3.3. Single-Crystal X-ray Diffraction Study

The data for single-crystal X-ray diffraction analyses were collected at 113 K on a ROD, Synergy Custom system (Rigaku Oxford Diffraction, Tokyo, Japan) equipped with mirror monochromated Mo-*K*α radiation. A suitable single crystal was mounted on a looped film (micromount) with Paraton-N. Data were processed using CrysAlisPro 1.171.39.43c (Rigaku Oxford Diffraction, Tokyo, Japan). The structures were solved using SHELXT [21] and the whole structure was refined against *F*^2^ with SHELXL-2018/3 [22]. All non-hydrogen atoms were refined anisotropically. Hydrogen atoms were located at idealized positions and were refined using a riding model with fixed thermal parameters. Crystal structure data (CIF, CCDC 2103614 for [Co^II^(hfac)_2_(PyBTM)_2_] and 2103615 for [Ni^II^(hfac)_2_(PyBTM)_2_]) can be obtained free of charge from the Cambridge Crystallographic Data Centre.

### 3.4. Powder X-ray Diffraction Study

Powder X-ray diffraction measurements were performed at room temperature using a MiniFlex600 diffractometer (Rigaku Corp., Tokyo, Japan) (Cu-*K*α radiation, *λ*= 1.5406 Å) operating at 40 kV/15 mA with a *K*β foil filter. The recorded diffraction pattern was analyzed by SmartLab Studio II (Rigaku Corp., Tokyo, Japan). The initial lattice parameters were obtained from the single-crystal data measured at 113 K, and were refined by the Pawley method.

### 3.5. Magnetic Measurements

The temperature dependence of the magnetic susceptibility of complexes was measured with a MPMS-7 SQUID magnetometer (Quantum Design Japan, Inc., Tokyo, Japan). Aluminum foil was used as a sample container, whose magnetic contribution was subtracted as background by measuring its own magnetic susceptibility in every measurement. The diamagnetic correction *χ*_dia_ for the sample was carried out with Pascal’s constants. *χ*_dia_: 7.41 × 10^−4^ cm^3^·K·mol^−1^ for [Co^II^(hfac)_2_(PyBTM)_2_]; 7.40 × 10^−4^ cm^3^·mol^−1^ for [Ni^II^(hfac)_2_(PyBTM)_2_].

### 3.6. DFT Calculations

The calculations were carried out using the Gaussian 16 Revision C.01 program package [23]. The geometry of each complex was extracted from the crystallographic data. Single-point calculations were performed assuming either sextet or doublet states for [Co^II^(hfac)_2_(PyBTM)_2_] and quintet or singlet states for [Ni^II^(hfac)_2_(PyBTM)_2_], at the uB3LYP level of theory with the LANL2DZ (Hay-Wadt ECP) basis set for the metal atoms and the 6-31G(d) basis set for the other atoms [24,25,26]. The intramolecular exchange interactions were considered within the approximate spin-projection (AP) method proposed by Yamaguchi and co-workers [27,28,29]. Discussion on the difference between the calculated *J*_M–R_/*k*_B_ values and the experimentally obtained ones is provided in the Supplementary Materials.

## Data Availability

The data presented in this study are available on request from the corresponding author.

## Supplementary Materials

Discussion on *J*_M–R_/*k*_B_ values; Figure S1. Observed and calculated powder X-ray diffraction profiles for compounds; Figure S2. Crystal structure of [Ni^II^(hfac)_2_(PyBTM)_2_]; Figure S3. Diffuse reflectance spectra of compounds; Table S1. Crystallographic data of complexes.

## Sample Availability

Samples of the compounds are available from the authors.
