# Supplementary material for: Structural and Magnetic Studies on Nickel(II) and Cobalt(II) Complexes with Polychlorinated Diphenyl(4-pyridyl)methyl Radical Ligands
Source: Molecules. 2021 Sep 15;26(18):5596. doi: 10.3390/molecules26185596 (PMC8469047; doi:10.3390/molecules26185596)
Supplement: Supplementary file 1 [file molecules-26-05596-s001.zip › molecules-1375427-supplementary.pdf]

molecules

Supporting Information

Structural and Magnetic Studies on Nickel(II) and Cobalt(II) Complexes with Polychlorinated Diphenyl(4-pyridyl)methyl Radical Ligands

Ryota Matsuoka[1,2], Tatsuhiro Yoshimoto[3] and Yasutaka Kitagawa,[4] Tetsuro Kusamoto[1,2,5]*

[1] Institute for Molecular Science, 5-1 Higashiyama, Myodaiji, Okazaki, Aichi 444-8787, Japan
[2] SOKENDAI (The Graduate University for Advanced Studies), Shonan Village, Hayama, Kanagawa 240-0193, Japan
[3] Department of Materials Chemistry, Ryukoku University, Otsu, Shiga, 520-2194, Japan
[4] Division of Chemical Engineering, Department of Materials Engineering Science, Graduate School of Engineering Science, Osaka University, Toyonaka, Osaka 560-8531, Japan
[5] JST-PRESTO, 4-1-8, Honcho, Kawaguchi, Saitama 332-0012, Japan
* Correspondence: kusamoto@ims.ac.jp

Table S1. Crystallographic data of $[Co^{II}(hfac)_2(PyBTM)_2]$ and $[Ni^{II}(hfac)_2(PyBTM)_2]$.

	$[Co^{II}(hfac)_2(PyBTM)_2]$	$[Ni^{II}(hfac)_2(PyBTM)_2]$
CCDC Number	2103614	2103615
Empirical formula	$C_{46}H_{14}Cl_{16}F_{12}N_2O_4Co$	$C_{46}H_{14}Cl_{16}F_{12}N_2O_4Ni$
Fw / g mol^{-1}	1512.72	1512.50
Crystal system	Triclinic	Triclinic
Space group	P-1	P-1
Crystal size / mm	0.23×0.15×0.09	0.33×0.17×0.09
Temperature / K	113	113
a / Å	9.0739(2)	9.0471(2)
b / Å	14.5648(3)	14.6129(3)
c / Å	20.9453(4)	20.8041(4)
α / °	97.212(2)	97.547(2)
β / °	94.578(2)	94.806(2)
γ / °	90.621(1)	90.560(2)
V / Å^3	2736.85(10)	2716.38(10)
ρ_{calced} / g cm^{-3}	1.836	1.849
λ / Å	0.71073	0.71073
μ / mm^{-1}	1.183	1.233
R_{int}	0.0411	0.0901
R_1[a]	0.0549	0.1351
wR_2[b]	0.1382	0.3543
GoF[c]	1.073	1.108

[a] $R_1 = \Sigma||F_o|-|F_c||/\Sigma|F_o|$ (I>2σ(I)), [b] $wR_2 = [\Sigma(w(F_o^2-F_c^2)^2/\Sigma w(F_o^2)^2]^{1/2}$ (I>2σ(I)), [c] GoF = $[\Sigma(w(F_o^2-F_c^2)^2/\Sigma(N_r-N_p)^2]$.

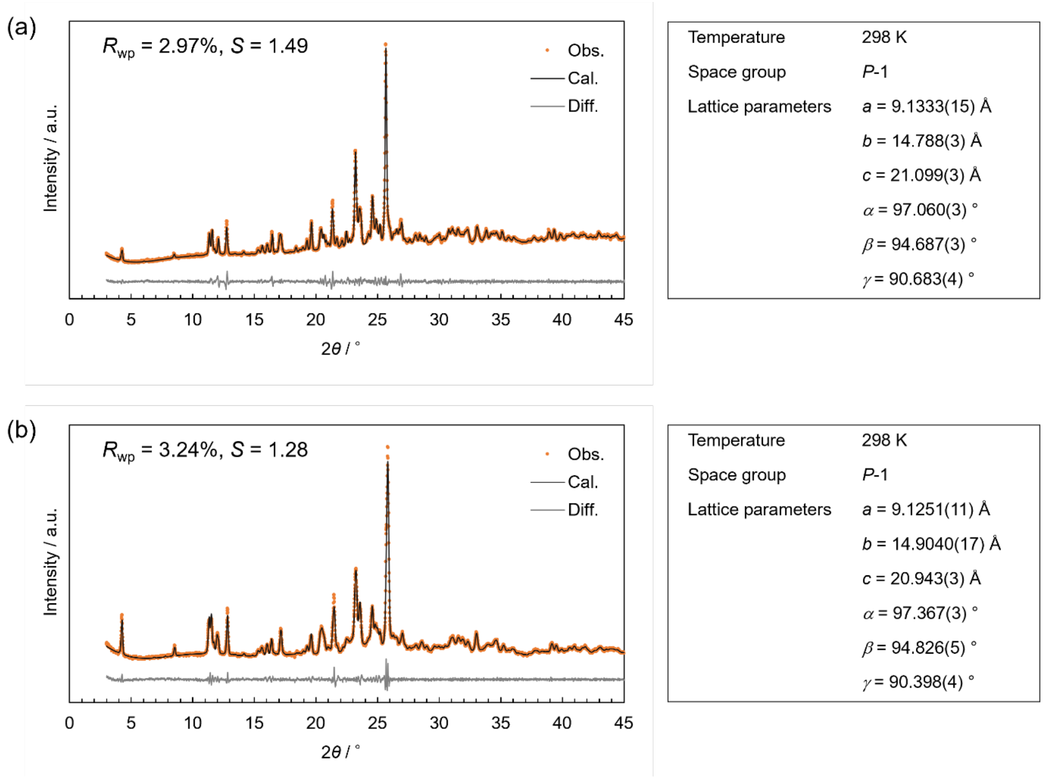

Figure S1. Observed (orange dot) and calculated (black line) powder X-ray diffraction profiles for the Pawley refinement of (**a**) $[Co^{II}(hfac)_2(PyBTM)_2]$ and (**b**) $[Ni^{II}(hfac)_2(PyBTM)_2]$ (298 K). The bottom gray curve represents the difference plot on the same intensity scale. The crystallographic data determined by the powder X-ray diffraction analyses are summarized in the right boxes.

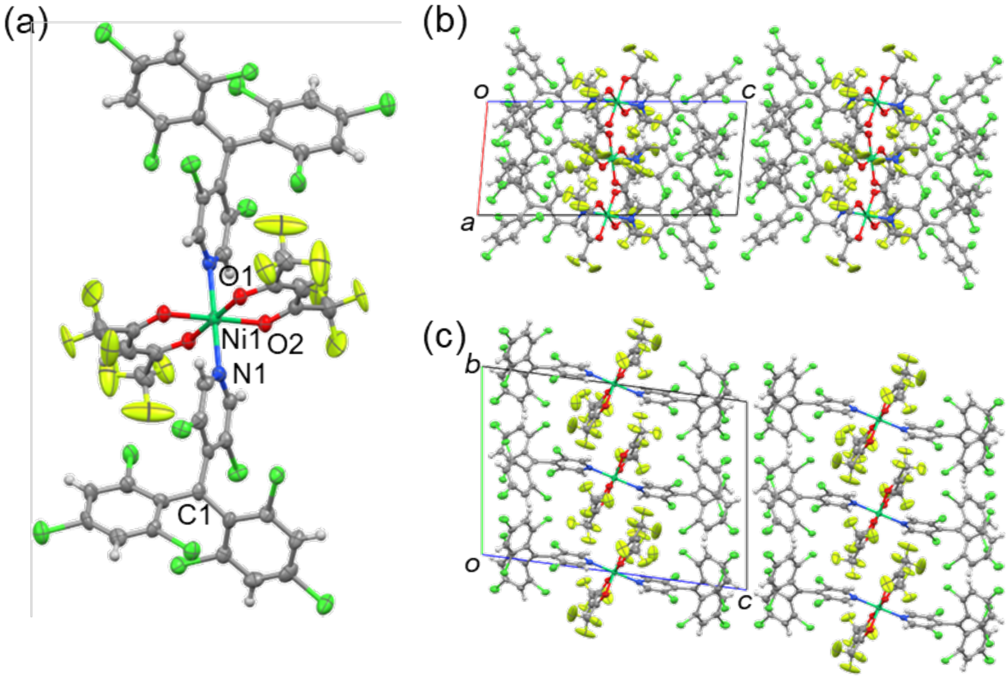

Figure S2. (**a**) Molecular structure of [Ni^{II}(hfac)$_2$(PyBTM)$_2$] in the crystal. (**b**) Crystal structure viewed along the *b*-axis and (**c**) the *a*-axis. The disorder of trifluoromethyl groups is omitted for clarity.

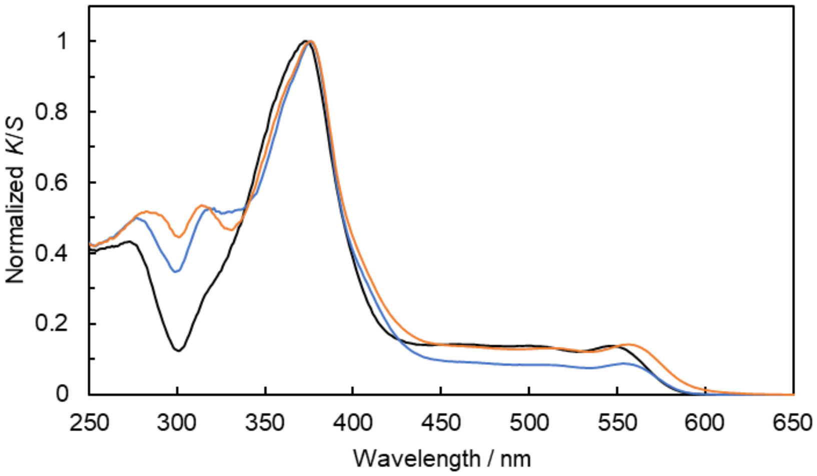

Figure S3. Diffuse reflectance spectra of [Co^{II}(hfac)$_2$(PyBTM)$_2$] (orange), [Ni^{II}(hfac)$_2$(PyBTM)$_2$] (blue), and PyBTM (black) crystals dispersed in KBr.

Both of the complexes had absorption bands in the UV region (250–350 nm) in addition to those corresponding to PyBTM, which are assignable to the π–π* and n–π* transitions of the hfac ligands. This indicates the coexistence of PyBTM and hfac moieties in the complexes.

Discussion on difference between calculated $J_{M\text{–}R}/k_B$ values and experimentally obtained ones

The calculated $J_{M\text{–}R}/k_B$ values reproduce the experimentally obtained value in the Ni(II) complex within the used level of theory, while the former are a little smaller than the latter in the Co(II) complex. The difference is considered to originate in the B3LYP hybrid functional set. It has been reported that B3LYP sometimes overestimates the stability of the anti-ferromagnetic state, especially for the through-space exchange interaction, due to a deficiency in the ratio of the Hartree-Fock exchange term.[1-3] The overstabilization of the anti-ferromagnetic state, for the ferromagnetic molecules in this paper, leads to the underestimation of the positive J values. This problem can be overcome by tuning a ratio between the Hartree-Fock and DFT exchange terms. The approach, however, digresses from the main topic of this paper. Further analyses and methodological improvements, therefore, were not performed here.

References

1. Kitagawa, Y.; Soda, T.; Shigeta, Y.; Yamanaka, S.; Yoshioka, Y.; Yamaguchi, K. Improvement of the Hybrid Density Functional Method from the Viewpoint of Effective Exchange Integrals. *Int. J. Quantum Chem.* **2001**, *84*, 592–600.
2. Kitagawa, Y.; Kawakami, T.; Yamaguchi, K. Instability of a System and Its Estimation in Terms of the Hybrid Density Functional Theory Method: A Magnetic Effective Density Functional (MEDF) Approach. *Mol. Phys.* **2002**, *100*, 1829–1838.
3. Soda, T.; Kitagawa, Y.; Onishi, T.; Takano, Y.; Shigeta, Y.; Nagao, H.; Yoshioka, Y.; Yamaguchi, K. Ab Initio Computations of Effective Exchange Integrals for H-H, H-He-H and Mn_2O_2 Complex: Comparison of Broken-Symmetry Approaches. *Chem. Phys. Lett.* **2000**, *319*, 223–230.
